# Single-Cell Transcriptome Comparison of Bladder Cancer Reveals Its Ecosystem

**DOI:** 10.3389/fonc.2022.818147

**Published:** 2022-02-21

**Authors:** Yongxiang Luo, Tao Tao, Ran Tao, Guixiao Huang, Song Wu

**Affiliations:** ^1^ Institute of Urological Surgery, The Third Affiliated Hospital of Shenzhen University, Shenzhen University, Shenzhen, China; ^2^ Shenzhen Following Precision Medical Institute, The Third Affiliated Hospital of Shenzhen University, Shenzhen University, Shenzhen, China; ^3^ Department of Urology, The Affiliated South China Hospital of Shenzhen University, Shenzhen University, Shenzhen, China

**Keywords:** bladder cancer, single-cell sequencing, microenvironment, N-glycan biosynthesis, different grades

## Abstract

Bladder carcinoma (BLCA) is a highly heterogeneous disease, and the underlying biological behavior is still poorly understood. Here, single-cell RNA sequencing was performed on four clinical samples of different grades from three patients, and 26,792 cell transcriptomes were obtained revealing different tumor ecosystems. We found that N-glycan biosynthesis pathway was activated in high-grade tumor, but TNF-related pathway was activated in cystitis glandularis. The tumor microenvironment (TME) of different samples showed great heterogeneity. Notably, cystitis glandularis was dominated by T cells, low-grade and high-grade tumors by macrophages, while TME in patient with high-grade relapse by stromal cells. Our research provides single-cell transcriptome profiles of cystitis glandularis and BLCA in different clinical states, and the biological program revealed by single-cell data can be used as biomarkers related to clinical prognosis in independent cohorts.

## Introduction

Cystitis glandularis (CG) usually occurs in chronic inflammation, with epithelial hyperplasia and metaplasia. The nest-like hyperplasia of epithelial cells in the lamina propria of the mucosa is called von Brunn’s nests (1). Although it is widely believed that cystitis glandularis is a benign lesion, patients with intestinal metaplasia and urinary tract accumulation are still at risk of developing bladder cancer (2). Bladder cancer is the most common malignant tumor of the urinary system, and urothelial cancer is the most common pathological type. About 75% are diagnosed as nonmuscle-invasive bladder cancer (NMIBC), of which 50%–70% will recur after surgery and 10%–20% will progress to muscle-invasive bladder cancer (MIBC) (3, 4). NMIBC papillary lesions are divided into urothelial papilloma, papillary urothelial neoplasm of low malignant potential (PUNLMP), low-grade (LG) papillary urothelial carcinoma, and high-grade (HG) papillary urothelial carcinoma (5). The higher the grade, the higher the malignancy. At present, the mechanism of the development of BLCA is not clear, and whether CG is a precancerous lesion is also controversial. Therefore, it is necessary to conduct an in-depth discussion on the occurrence and development mechanism of BLCA and the relationship between BLCA and CG from the cellular levels.

The emergence of new treatments for diseases is inseparable from a deep understanding of the identity and function of diseased tissue cells. The progress of single-cell RNA sequencing (scRNA-seq) provides a method for people to explore the genetic and functional heterogeneity of complex biological systems at a cellular resolution. The technology can evaluate tens of thousands of cells at the same time and can reveal the regulation, communication, and interaction between cells (6). Since the potentially different components of bulk transcription profiles hinder direct comparison of different cell types, single-cell transcription profiles provide a suitable alternative method that makes direct comparisons between the same cell types possible. However, the current single-cell studies of bladder cancer mostly focus on the normal bladder (7) and tumor microenvironment (TME) (8), and there is still a lack of comparative studies on the single-cell level of bladder cancer with different grades and before and after recurrence.

We performed scRNA-seq on three bladder cancer tissues and one CG tissue and obtained transcriptome profiles of 26,792 cells. Through comparative analysis between different samples, we comprehensively described the expression characteristics of malignant epitheliums and immune cells, as well as the dynamic changes of cell percentages, and the heterogeneity of cell subtypes. This study provides new biological knowledge about the cell composition of CG and the heterogeneity of different grades of bladder cancer.

## Materials and Methods

### Sample Collection

The four bladder samples used in this study were collected from patients undergoing transurethral resection of bladder tumor (TURBT) surgery in Luohu District People’s Hospital. All experiments were conducted after approval by the Ethics Committee Board of Luohu District People’s Hospital, and informed consent forms were also signed by the participating patients.

### Tissue Disassociation

After the fresh tissue samples were surgically removed, they were stored in GEXSCOPETM Tissue Preservation Solution (Singleron, Cologne, Germany) and taken back to the laboratory for further processing. First, the tissue samples were washed by using Hanks Balanced Salt Solution (HBSS), and then cut them into 1~2 mm pieces. The small tissue pieces were transferred to a 15-ml centrifuge tube and digested using GEXSCOPETM Tissue Dissociation Solution (Singleron) under shaking conditions at 37°C for 15 min. After the digestion, the samples were filtered with 40 µm sterile strainers, and then centrifuged at 1,000 rpm at 4°C for 5 min. The supernatant was removed, and the cells were resuspended with 1 ml PBS, then added 2 ml GEXSCOPETM red blood cell lysis buffer (Singleron) and incubated for 10 min to lyse the red blood cells. Finally, the single cell suspension was collected after resuspension with PBS.

### Single-Cell RNA Sequencing

According to the Singleron GEXSCOPER operation steps, the GEXSCOPER microfluidic chip was used to capture the single cell suspension with a concentration of 1~3×105 cells/mL. There was a magnetic bead paired with a cell in each microwell, and each magnetic bead had a unique cell label and multiple molecular labels [unique molecular identifier (UMI)] for capturing RNA, so when the RNA is released from the captured cells, it can hybridize to the magnetic beads. The magnetic beads were then collected into a 1.5-ml Eppendorf tube for reverse transcription, using GEXSCOPER Single-Cell RNA Library Kit to perform cDNA synthesis on single cells (9). After cDNA synthesis and PCR amplification, Qubit (Thermo, Waltham, MA, USA) for cDNA Quantification and Fragment Analyzer were used to determine the cDNA fragment size. After a series of steps, including fragmentation, adapter ligation, purification, PCR amplification, size selection, and QC, the libraries were constructed. The library was lastly sequenced with 150 bp paired end reads on the Illumina HiSeq X platform.

### Sequencing Data Processing

The original gene expression matrix data were generated using the CeleScope (https://github.com/singleron-RD/CeleScope) software. CeleScope is a single-cell data processing software developed by Singleron that can execute a series of standard analysis procedures and can finally display the gene expression matrix required for downstream analysis. Briefly, after using fastqc (version 0.11.7) and cutadapt (version 1.17) to quality control and filter the data, reads were compared with the reference genome GRCh38 with ensemble version 93 gene annotation using STAR (version 2.6.1b). Finally, featureCounts (version 1.6.2) was used to output the gene count matrix.

Subsequently, the gene expression matrix was imported into R (version 4.0.3) software, and the Seurat (version 4.0.1) package was used for downstream analysis. The processing flow of each sample was basically the same, but the standard for filtering low-quality cells for each sample was different. In brief, in the LH0826 sample, the cells with genes <200 or >4,500 or UMIs derived from mitochondria >75% were considered low-quality cells; the LHBC0803 sample was cell with genes <200 or >5,000 or UMIs derived from mitochondria >55%; the LHBC0911 sample was cells with genes <200 or >3,500 or UMIs derived from mitochondria >60%; the LHBC0917 sample was cells with genes <200 or >5,000 or UMIs derived from mitochondria >50%. After removing low-quality cells, the count data were firstly normalized by using the NormalizeData function. The FindVariableFeatures function was then used to select 2,000 intercellular variant genes, and ScaleData function was used to normalize the expression data of all genes before these variant genes were mapped to the low-dimensional subspace using the principal component analysis function RunPCA. The FindNeighbors function was used to construct the common nearest-neighbor graph with the Euclidean distance metric in ten principal component low-dimensional subspaces. After using the FindClusters function (res = 0.5) to cluster the cells, the RunUMAP function visualized the clustered cells. All implementation steps referred to the website tutorial (https://satijalab.org/seurat/).

### Integrated Analysis

To compare the cell types of different samples, the FindIntegrationAnchors function and the IntegrateData function were used to identify anchors. These anchors were then used to integrate multiple datasets together, which will automatically correct the batches of all samples to generate an integrated expression matrix containing 2,000 genes in all cells.

After the RNA expression matrix integrated by all samples was performed nonlinear dimensionality reduction, clustering, and visualization, the known marker genes (10, 11) were used to annotate the cell types of each cluster (**Supplementary Table S1**).

### CNV Inference

According to the previously described methods (11), the R package infercnv (version 1.2.1) (cutoff = 0.1) was used to estimate the CNV of epithelial cells, and stromal cells were considered nonmalignant cells as a reference. The algorithm of infercnv considered that gene expression in the region of the copy number variation was either too high or too low compared with normal chromosomes. A sliding window including 100 genes was used to average the expression of these genes on each chromosome. The CNV score of each cell was defined as the mean square of the deviation in the genome (mean((cnv-1)^2)). Finally, the first two clusters were identified as malignant epithelium (*n* = 10,339).

### Analysis of Malignant Epithelial Cells

Malignant epithelium (*n* = 10,339) obtained by infercnv analysis was subset to further analysis. We used the default parameters of the FindMarkers function in Seurat to identify differentially expressed genes in samples of different clinical conditions (CG vs. cancer, LG vs. HG, and HG vs. HG relapse), and the criteria for upregulated gene selection were *p* < 0.05 and avg_log2FC>0.5. Since some genes may not be fully detected during the sequencing process, leading to their low expression, the main focus was on upregulated genes when analyzing the activated pathways. Besides, activated gene signatures mainly consist of up-regulated differential genes.

### Gene Enrichment Analysis

The enrichment analysis was performed on all the differentially expressed genes. The Kyoto Encyclopedia of Genes and Genomes (KEGG) annotations were collected from previous work (12), and GO annotations were downloaded from NCBI (ftp://ftp.ncbi.nlm.nih.gov/gene/DATA). GSEA analysis was performed using the R package clusterProfiler (version 3.18.1), and the activation pathways standard was *p*-value <0.05 and enrichment score >0.5. In addition, the DAVID website (https://david.abcc.ncifcrf.gov/) and R package qusage (version 2.24.0) were also used to further search for activated pathways according to the previous methods (13).

### Survival Analysis

The differentially activated gene sets obtained by gene enrichment analysis were used as the gene signatures of the sample for survival analysis. We downloaded the TCGA BLCA expression data and clinical data from the database (https://xenabrowser.net/datapages/). The average expression level of N-glycan biosynthesis signatures was calculated for each TCGA BLCA sample, and then the TCGA samples were divided into two groups with high and low expression. The survival analysis was performed by R package survival (version 3.2.7). Log-rank test was used to detect the significance of the difference between the two groups, and *p* < 0.05 was considered a significant difference.

### Analysis of Nonepithelial Cells

Nonepithelial cells including immune and stromal cells (*n* = 3,496) were subset and reclustered according to the standard procedures, using parameters (features = 2,000, npcs = 30, res = 0.8). The resulting 10 clusters were annotated with different cell types using a list of known markers (11). We then evaluated the composition of different cell types in the tumor microenvironment of each sample.

Normalizing, scaling, and clustering were performed according to the methods described above. T cells (*n* = 365) were divided into 4 groups using parameters (features = all genes, npcs = 30, res = 0.5), macrophages (*n* = 853) were divided into 5 groups using parameters (features = all genes, npcs = 30, res = 0.3), endothelial cells (*n* = 548) were divided into 3 groups using parameters (features = all genes, npcs = 30, res = 0.5), and fibroblasts (*n* = 176) were divided into 5 groups using parameters (features = all genes, npcs = 30, res = 0.5). The FindAllMarkers function was used to identify the markers of different clusters.

### Trajectory Analysis

We used the R package monocle (version 2.18.0) to carry out single-cell trajectory analysis, and the dimensionality reduction method used was DDRTree, which was a new manifold learning algorithm. All implementation details involved in trajectory analysis referred to the standard process (http://cole-trapnell-lab.github.io/monocle-release/docs/). Briefly, we constructed a monocle object from the seurat object, obtained a list of differential genes using the dispersionTable function, and placed the cells in the pseudotime trajectory using the orderCells function based on the selected highly variable genes.

### Analysis of Oligosaccharide Transferase Inhibition

The human bladder cancer cell line T24 (ATCC) and mouse bladder cancer cell line MBT2 (ATCC) were cultured in RPMI-1640 medium (GIBCO, Waltham, MA, USA) with 10% FBS and 1% penicillin-streptomycin. Next, T24 and MBT2 cells (1 × 104) were seeded in 96-well plates, respectively, treated with different concentrations of NGI-1 and cisplatin (DDP) for 24 h, and then counted with CCK8 following the standard protocol. The significance values of different treatment groups were calculated by Student’s *t*-test.

### Expression of OST-Related Genes Detected by q-PCR

Bladder cancer cell lines RT4, T24, and EJ were seeded in six-well plates. Total RNA was extracted using MolPure^®^ Cell/Tissue Total RNA Kit (Yeasen, Shanghai, China, 19221ES50), and RNA concentration was determined by NanoDrop™ One/OneC Spectrophotometer. RNA was reverse transcribed using Hifair^®^ III 1st Strand cDNA Synthesis SuperMix for qPCR (Yeasen, Shanghai, China, 11141ES60), and finally real-time PCR analysis was performed using a 7500 Real-Time PCR Detection System (Applied Biosystems, Waltham, MA, USA). The data were analyzed using the delta delta threshold cycle (DDCt) method for relative quantification of gene expression. A *t*-test was used to calculate the significance *p*-value between low-grade and high-grade bladder cancer cell lines.

### Immunofluorescence Analysis

Paraffin-embedded samples were dewaxed, hydrated, heat treated for antigen repair, incubated with primary antibody, and added with corresponding fluorescent secondary antibody. The primary and secondary antibodies were washed off and incubated with the second primary antibody and the corresponding fluorescent secondary antibody. DAPI-counterstained (G1012, Servicebio, Wuhan, China) nuclei were incubated at room temperature for 10 min. Spontaneous fluorescence quenching reagent (G1221, Servicebio) was added and incubated for 5 min to quench the autofluorescence of tissues. After the slices were slightly dried, they were sealed with antifade mounting medium (G1401, Servicebio). The sections were observed under a fluorescence microscope, and the images were analyzed by CaseViewer (version 2.4.0).

The following antibodies were used to detect the corresponding proteins: CD14 (GB14023, dilution 1:100, Servicebio), CD68 (GB113150, dilution 1:200, Servicebio), CD3 (GB14176, dilution 1:100, Servicebio), and CD8 (GB13068, dilution 1:200, Servicebio). The used fluorescent secondary antibodies include CY3 goat anti-mouse antibody (GB21301, dilution 1:300, Servicebio) and 488 goat anti-rabbit antibody (gb25303, dilution 1:400, Servicebio).

### Statistical Analysis

All statistical analyses were implemented in R software (version 4.0.3), and statistical methods for calculating *p*-values were detailed in the figure legends or main text.

## Results

### Single-Cell Transcriptome Profile of Bladder Samples

**Figure 1:**
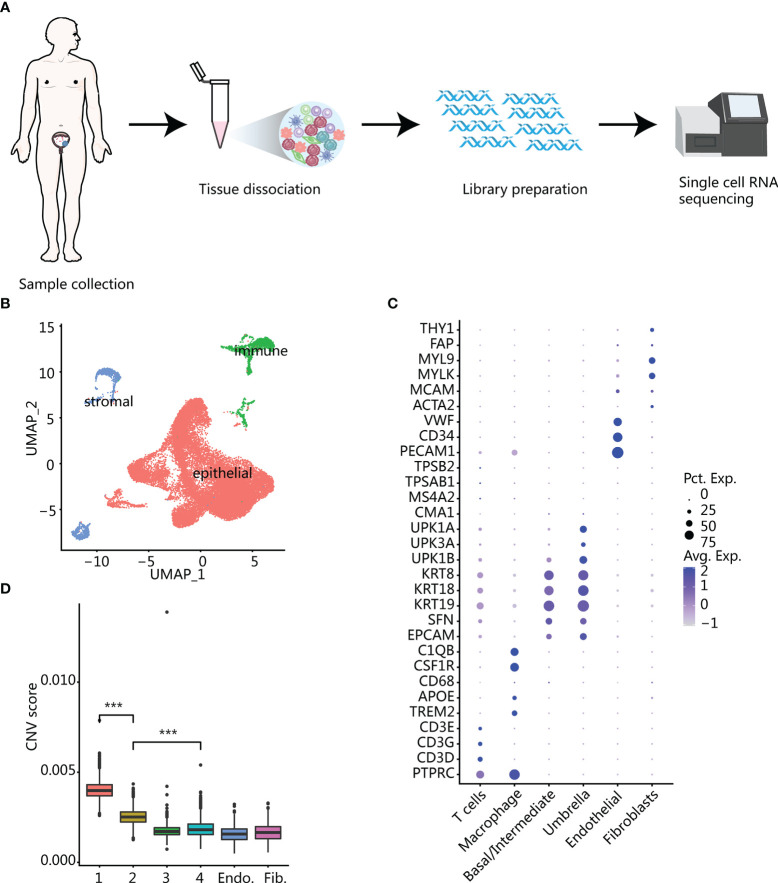
Overview of all cells in bladder samples. **(A)** The experimental process of single-cell RNA sequencing workflow. **(B)** UMAP plot of the general cell cluster. One point represents one cell, and different colors represent different cell types. **(C)** Dot plot of marker gene expression in different cell types. The size of the dot indicates the percentage of marker gene expression, and the color of the dot indicates the average expression of marker genes. **(D)** CNV score inferred by infercnv in different epithelial cell clusters and endothelium and fibroblasts. Clusters 1 and 2 with the highest scores were identified as malignant epithelium. ^***^ *p* < 0.001 using two-sided unpaired Wilcoxon rank sum test.

We performed single-cell sequencing on four bladder samples from three patients (one case of cystitis glandularis (CG), one case of low-grade (LG) bladder cancer and two samples of high-grade (HG) bladder cancer before and after recurrence in the same patient) (**Supplementary Table S1**). According to the scRNA-seq experimental design, we need to get viable single cells from fresh tissue samples which were resected from patients (**Figure 1A**). After standard data preprocessing procedures and quality control, we obtained 26,792 cells’ RNA transcription profiles. By normalizing gene expression profiles, principal component analysis, and graph-based clustering methods, we identified 17 cell populations (**Supplementary Figure S1A**). Using canonical cell-type markers, we annotated the obtained cell populations into three major categories: immune (T and macrophage), stroma (fibroblasts and endothelial cells), or epithelial cells (base and umbrella cells) (11) (**Figures 1B, C**). Because cancer was related to genome copy number variation (CNV), we used transcriptome data to infer the CNV of the cells, so as to judge whether the epithelial cells were normal or malignant (14, 15). Compared with reference fibroblasts and endothelial cells, malignant epithelium showed large-scale genomic variation, and the inferred CNV was roughly the same as that of classic bladder cancer genomic variation (16), including chromosome 8q amplification and 8p deletion (**Supplementary Figure S1B**). We calculated the average CNV scores and compared them with that of the control group; epithelial clusters 1 and 2 had higher CNV scores, which were inferred to be malignant cells (**Figure 1D**). Most of the cells derived from samples of CG were inferred to be nonmalignant cells. It is worth noting that a small number of epithelial cells from CG have also been inferred to be malignant epithelium, indicating that CNVs are also present in normal epithelial cells in an inflammatory state. In addition, compared with malignant cells, normal epithelial cells had a higher genetic complexity (**Supplementary Figure S1C**).

**Figure 2:**
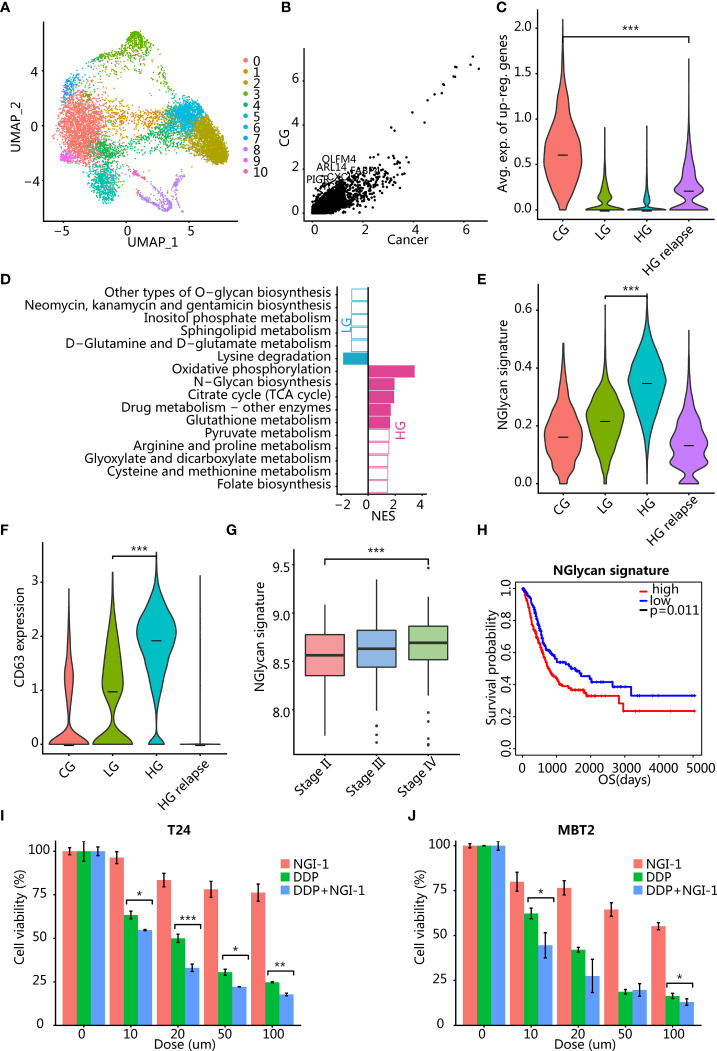
Analysis of differential gene expression between malignant epitheliums in different clinical states. **(A)** UMAP plot of 10,339 malignant epithelial cells. Color indicates different cell clusters. **(B)** Scatter plot showed that a small number of genes were upregulated in CG. **(C)** Violin plots showing the expression level of the GC signature (average expression of CG upregulated genes) in different clinical states. ^***^ *p* < 0.001 using two-sided unpaired Wilcoxon rank sum test. **(D)** Bar plots showing the pathway differentially activated between LG and HG. The color indicates significance *p* < 0.05, and the *x*-axis is the enrichment fraction. **(E)** Violin plots showing the expression level of N-glycan signature (the average expression of genes enriched into this pathway) in different clinical states. ^***^ *p* < 0.001 using two-sided unpaired Wilcoxon rank sum test. **(F)** Violin plots showing the expression level of CD63 in epithelial cells of different clinical states. ^***^ *p* < 0.001 using two-sided unpaired Wilcoxon rank sum test. **(G)** Boxplots showing the distribution of N-glycan signature (the average expression of genes enriched into this pathway) in TCGA BLCA cohort pathological stages. ^***^ *p* < 0.001 using two-sided unpaired Wilcoxon rank sum test. **(H)** Kaplan-Meier analysis of N-glycan signature with overall patient survival in TCGA dataset. *p* = 0.011 using log-rank test. **(I, J)** Therapeutic response to inhibition of N-glycan pathway activity in T24 **(I)** and MBT2 **(J)** cells. ^*^ *p* < 0.05, ^**^ *p* < 0.01, ^***^ *p* < 0.001 using two-sided unpaired Student’s *t*-test. T24 and MBT2 cells were treated with NGI-1 or cisplatin (DDP) or combined DDP and NGI-1. Error bars indicate addition and subtraction of standard deviation.

The malignant epithelial cells were taken out and reclustered to obtain 11 clusters (**Figure 2A**). Most of the clusters of malignant epithelial cells came from multiple patients, but high-grade patients had a larger proportion of malignant epithelial cells, suggesting that malignant epithelial cells had greater heterogeneity (**Figure 2A**; **Supplementary Figure S1D**).

### TNF Signaling Pathway Activated in CG

We hypothesized that the signaling pathways activated in cystitis are mainly related to the inflammatory responses. We compared the transcriptome differences between malignant epithelium of patients with CG and malignant epithelial cells of cancer patients and found that most genes were downregulated in CG and only a few genes were upregulated in cystitis, such as OLFM4, PIGR, FABP4, TCIM, MUC4, DENND2C, ARL14, and CXCL8 (**Figures 2B, C**). Enrichment analysis confirmed our conjecture that differentially overexpressed genes in CG epithelial cells were mainly enriched in pathways related to immune activation and inflammatory response, including TNF signaling pathway and IL-17 signaling pathway. Tumor necrosis factor (TNF) pathway-related genes were significantly upregulated in CG (**Supplementary Figure S2B**), such as EDN1, CXCL2, PTGS2, CXCL1, IL1B, and MAP2K3 (**Supplementary Table S2**). The proinflammatory cytokine IL1B was produced by activated macrophages and T cells and induced the production of PTGS2 to synthesize prostaglandins to promote inflammation (17).

In addition, the terms of cellular response to tumor necrosis factor and extracellular exosome were enriched in upregulated genes of patients with CG (**Supplementary Table S1**). Since TNF was mainly produced by activated macrophages and T cells, we speculated that activated macrophages and T cells may secrete various proinflammatory cytokines through exosomes to cause an inflammatory response. We have further validated our conjecture by single-cell RNA sequencing data of macrophages and T cells. It was found that one macrophage cluster M4 highly expressed proinflammatory cytokine TNF, while T cells did not (**Supplementary Figures S2I, J**), suggesting that the inflammatory response was caused by the activation of macrophage subsets. Thus, inhibiting the activation of macrophages/T cells or applying TNF antagonists such as etanercept (18) may help the treatment of glandular cystitis.

### N-Glycan Biosynthesis Pathway Activated in HG

We compared cancer cells from patients with LG and HG bladder cancer to explain the difference in the transition from LG to HG and a total of 278 genes (NLG = 74, NHG = 204) that were significantly upregulated in LG or HG bladder cancer (*p* < 0.01) (**Supplementary Table S3**). LG bladder cancer overexpressed the apolipoprotein gene APOL1, which is related to cell death caused by autophagy (19). In addition, SP100 was also overexpressed in LG bladder cancer, which is associated with the ability to inhibit cell invasion (20, 21). The expression of these inhibitory genes controlled the growth of LG bladder cancer cells and improved clinical prognosis.

Among those HG bladder cancer overexpressed genes, we identified genes related to the N-glycan biosynthesis pathway (DAD1, DDOST, RPN1, RPN2, DPM3, DPM1, DPM2, ALG3, and MAN1A1) (**Figure 2D**). Several of these genes (DAD1, DDOST, RPN1, and RPN2) are an oligosaccharide transferase subunit protein, which plays an important role in protein localization, signal transduction, and intercellular communication through the glycosylation modification of the posttranslational protein (22). To further validate our findings, we verified the expression of major subunit genes of OST, a key enzyme of N-glycan biosynthesis pathway activated in high-grade bladder cancer, by q-PCR. The results showed that the expression of OST-related genes in high-grade bladder cancer cell lines T24 and EJ was higher than that in low-grade bladder cancer cell line RT4 (**Supplementary Figure S2H**). The abnormally high expression of RPN2 in a variety of cancer subtypes plays an important role in the drug resistance and invasion progression of cancer cells by regulating the N-terminal glycosylation of various proteins such as p-glycoprotein, CD63, and epidermal growth factor receptor (EGFR) (23–27). High expression of mannose transferase gene ALG3 can enhance proliferation, migration, and EMT ability (28), as well as stemness of cancer cells (29). We found that both N-glycan signature and CD63 were highest in HG bladder cancer (**Figure 2E, F** **;** **Supplementary Figure S2C**). In addition, we tested whether the N-glycan signature was clinically relevant in the TCGA bladder cancer (BLCA) transcriptome dataset and found that the N-glycan signature was significantly positively correlated with different stages (**Figure 2G**). Also, high expression of this signature was associated with poor prognosis of patients (**Figure 2H**, *p* = 0.011). Therefore, the use of oligosaccharide transferase inhibitors NGI-1 (30, 31) or aclacinomycin (32, 33) may be able to inhibit the progression of HG bladder cancer. To test our hypothesis, we used oligosaccharide transferase (a key enzyme of N-Glycan biosynthesis pathway) inhibitor NGI-1 combined with cisplatin (DDP) to explore the effects on bladder cancer cell lines T24 and MBT2. The results demonstrate that the inhibition of the N-glycan pathway in combination with chemotherapy significantly reduced cell viability in a dose-dependent manner (**Figures 2I, J**).

In addition, we compared the transcriptome differences before and after the recurrence of the same HG bladder cancer patient and found that HG patients before relapse still highly expressed the N-glycan biosynthesis pathway-related genes, although it was not significant (**Supplementary Figure S2D**). After recurrence, patients had high expression of genes related to cancer cell invasion and drug resistance, including regulation of cell growth [KIAA1109, IGFBP5 (34, 35), and PLCE1 (36)] and ABC transporters [ABCC5 and ABCC3 (37)] (**Supplementary Table S1**).

### Macrophage and T-Cell Infiltration Are Opposite in CG and Cancer Microenvironment

**Figure 3:**
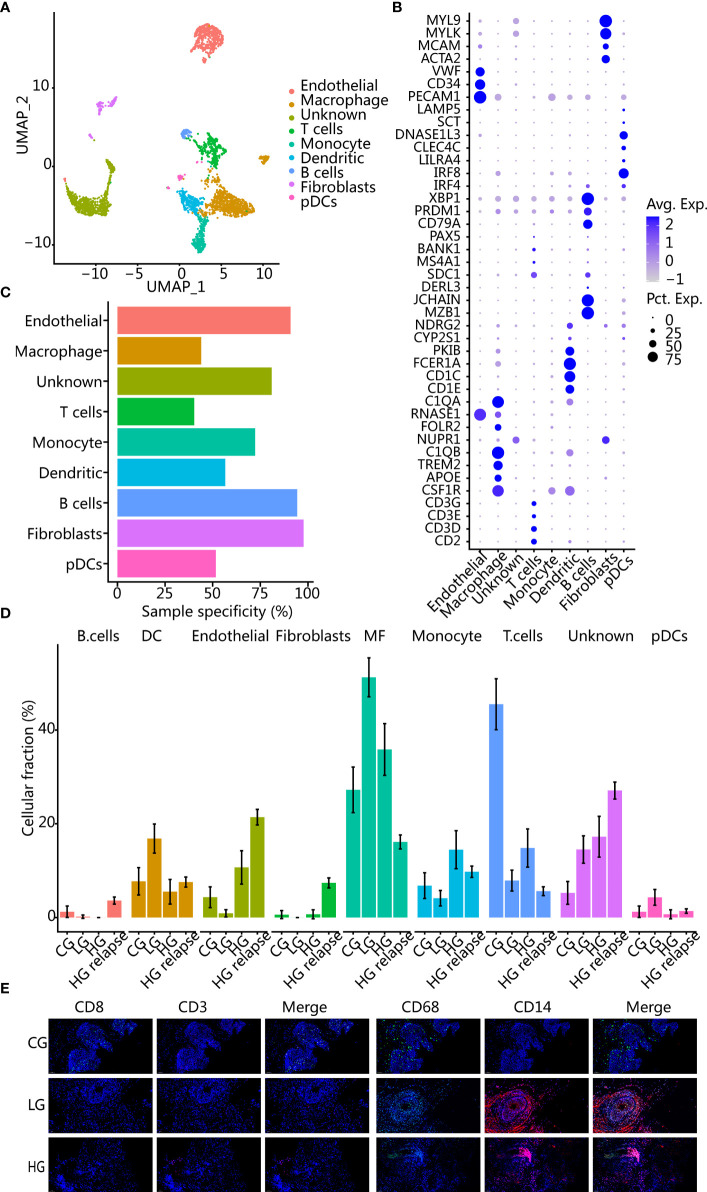
Changes of cell composition in microenvironment of different bladder samples. **(A)** UMAP plot of immune cells and stromal cells, showing different cell types. **(B)** Dot plot of marker gene expression in different cell types. **(C)** Sample specificity of each immune or stromal cell type. **(D)** Changes in the proportion of each cell type in samples with different clinical states. The error bar represents the 95% confidence interval of the cell proportion. **(E)** Immunofluorescence images showed changes in cellular components of CG, LG, and HG. CD8 and CD3 are T-cell markers; CD68 and CD14 are macrophage markers.

The tumor microenvironment (TME) plays a complex role in tumor progression, and we then evaluated the TME of different patients. Firstly, we reclustered the immune and stromal cells into 17 groups (**Supplementary Figure S2E**) and annotated them with known markers to obtain 8 cell types (**Figures 3A, B**). Among them, immune cells such as macrophages and T cells had low patient specificity. In contrast, stromal cells such as endothelial and fibroblast cell clusters showed high patient specificity (**Figure 3C**), which were enriched mainly in patients with HG relapse (**Figure 3D**).

All cancer patients had a higher proportion of macrophages than patient with CG, while patient with CG had a higher proportion of T cells than all cancer patients. Using immunohistochemistry staining further corroborated our findings (**Figure 3E**). After recurrence, T cells and macrophages were lower than before recurrence, while stromal cells were elevated, indicating that the patient with HG relapse had immunosuppression. In addition, we deconvolved the TCGA BLCA transcriptome data to obtain the proportion of immune cells in different samples and found that patients with a higher proportion of T cells had a better prognosis (*p* = 0.0088) (**Supplementary Figure S2F**). Patients with a higher proportion of macrophages had a poorer prognosis trend but not significant (*p* = 0.1942) (**Supplementary Figure S2G**).

### Memory T-Cell Phenotype Is Predominant at HG Relapse

**Figure 4:**
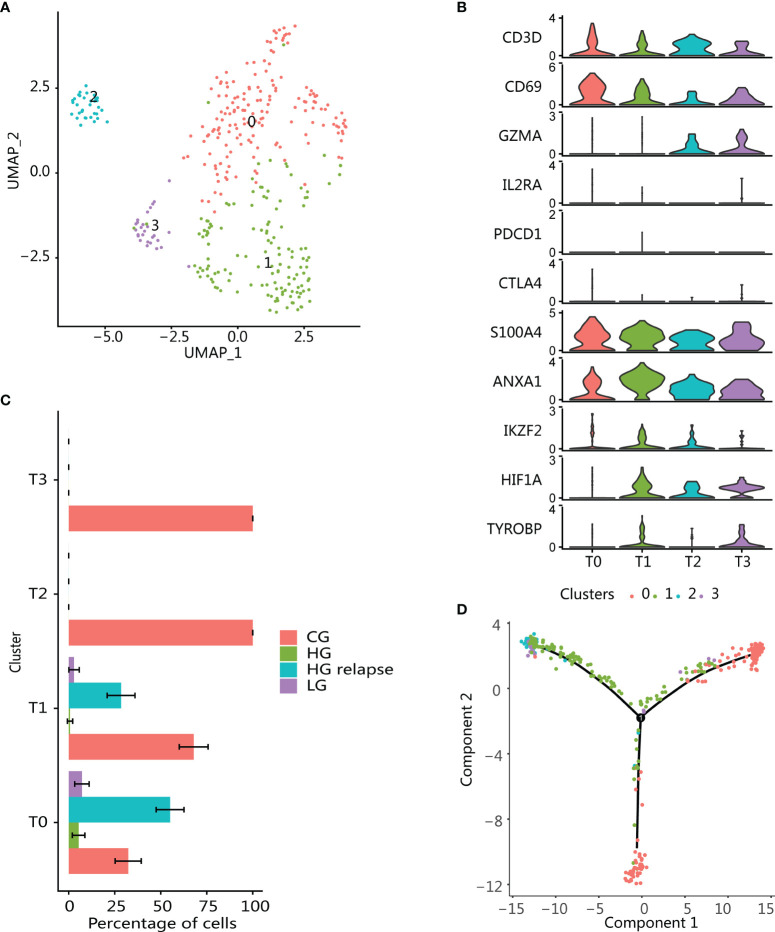
Enrichment of immune memory T-cell population in high-grade recurrent bladder cancer. **(A)** UMAP plot of all T cells. Color indicates T-cell subtype. **(B)** Violin plots showing the expression of marker genes in T-cell subtypes. **(C)** Changes in the proportion of each T-cell subgroup in samples with different clinical states. The error bar represents the 95% confidence interval of the cell proportion. **(D)** T-cell pseudotime development trajectory. Color indicates T-cell cluster.

T cells (*n* = 365) were reclustered in the same way as previously discussed, and 4 different T-cell populations were obtained (**Figure 4A**). In addition, DEG was also performed for each cluster (**Supplementary Figure S3B**). These cell clusters had obvious patient specificity (**Supplementary Figure S3A**). Three cell populations (T1, T2, and T3) were enriched in CG sample. The cell populations T2 and T3 were specifically derived from CG, and one cell population (T0) was enriched in HG relapse bladder cancer sample. Overall, the proportion of T cells was the highest in CG (**Figure 4C**), and there was no T-cell cluster that accounts for more in LG and HG bladder cancer samples. In addition, all T-cell clusters highly expressed T-cell markers (CD3D and CD69) and lacked the expression of inhibitory markers (PDCD1 and CTLA4) (**Figure 4B**), reflecting an activated T-cell state (38).

The cluster T0 was enriched in HG relapse state and overexpressed markers consistent with memory T-cell phenotype (CD69, S100A4, ANXA1) (**Figure 4B**) (39, 40). T-cell infiltration was mainly found in CG, including T-cell clusters T1, T2, and T3, and had similar enrichment pathway characteristics (**Supplementary Figure S3C**). These clusters all expressed memory T-cell markers, which might be derived from the differentiation of memory T cells. Moreover, T1 also expressed markers related to immunoregulatory functions, such as high expression of transcription factor HIF1A and low expression of Treg markers (IKZF2, IL2RA, and FOXP3), which were consistent with T helper (Th) cells (41). T2 was recognized as the effector T cell that highly expressed GZMA. T3 expression marker was consistent with the natural killer T-cell (NKT) phenotype, including the expression of NK cell marker TYROBP and effector T-cell marker GZMA (42). Using Monocle to perform pseudotime analysis of all T cells confirmed our conjecture. Starting from part of cluster T0, it progressed to cluster T2 and T3 at one end, part of cluster T0 at the other, while cluster T1 was the intermediate state along the axis of cluster T2 and T3. This reflected that memory T cells cannot only differentiate into effector T cells but also maintain their long-term survival through self-proliferation (**Figure 4D**).

### Immunosuppressive Macrophages Are Enriched in Tumor Microenvironment

**Figure 5:**
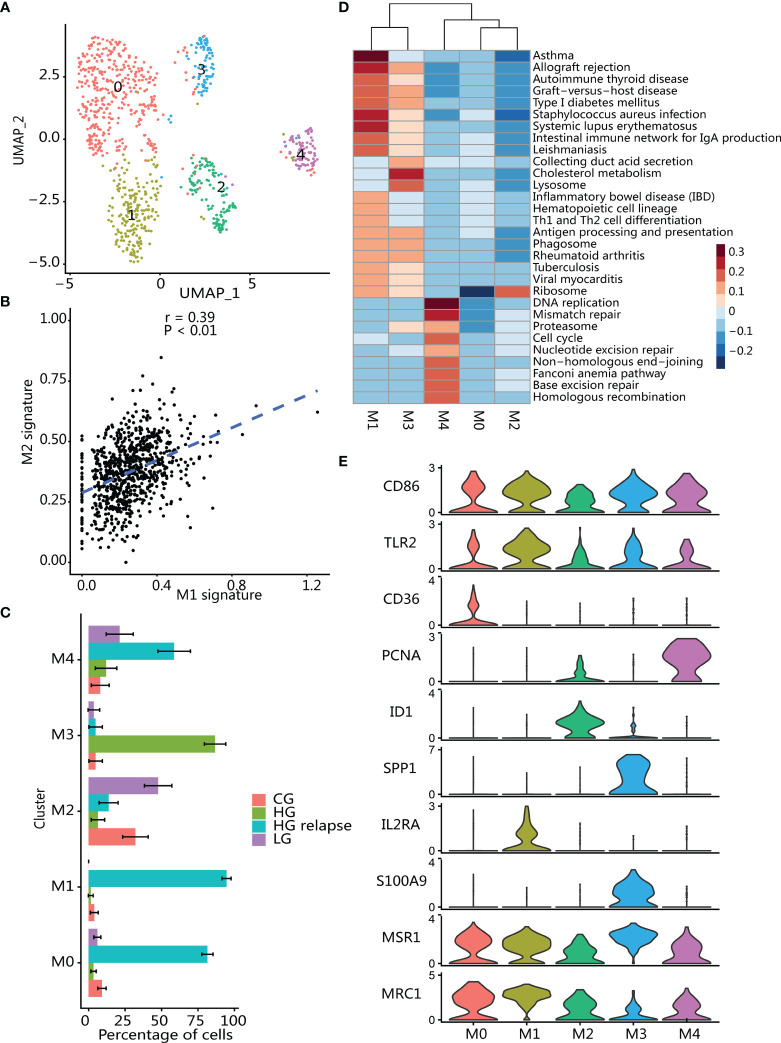
Immunosuppressive macrophages infiltrate in tumor microenvironment. **(A)** UMAP plot of macrophages; color indicates cell population subtypes. **(B)** Correlation between proinflammatory (average expression of M1 signatures) and reparative (average expression of M2 signatures) macrophages; each point represents one cell. Two-sided *p*-value was calculated for Pearson’s correlation coefficient. **(C)** Changes in the proportion of each macrophage subgroup in samples with different clinical states. The error bar represents the 95% confidence interval of the cell proportion. **(D)** Heatmap of differential activation pathways in macrophage clusters. **(E)** Violin plots showing the expression of marker genes in macrophage subtypes.

Macrophages are usually divided into proinflammatory and repairing types. Repairing macrophages can promote tumor growth and metastasis, while proinflammatory type is generally considered to have antitumor effects (43, 44). We found that proinflammatory type (CD86 and TLR2) and repairing type (MSR1 and MRC1) signatures were activated in all macrophage clusters (40) (**Figure 5E**), and two macrophage type signatures showed a positive correlation (**Figure 5B**).

Macrophages (*n* = 853) were reanalyzed in the same way as T cells and divided into 5 clusters **(** **Figure 5A**), and then differential expression analysis for each cluster was performed (**Supplementary Figure S3E; Supplementary Table S4**). Clustering analysis found that these clusters had high patient specificity (**Supplementary Figure S3D**), and then we calculated the proportion of each macrophage cluster in the patients to which it belonged (**Figure 5C**). Clusters M0, M1, and M4 were significantly enriched in patient with HG relapse. Cluster M0 highly expressed immunosuppressive and angiogenic phenotype-related markers (SLC40A1, NRP1, and CD36) (45–47) and promote tumor progression associated with markers (CD163 and CD163L1) (48, 49). In addition, cluster M0 also highly expressed CCL4, a chemokine that can promote tumorigenesis and progression by recruiting regulatory T cells and protumorigenic macrophages (50). Cluster M1 was significantly enriched in immune-related pathways such as Th1 and Th2 cell differentiation and antigen processing and presentation (**Figure 5D**), and high expression antigen presents HLA-related genes and inflammation-related genes (C3, IL2RA, LY86, CX3CR1) (51–53), and macrophages can promote tumor cell growth and metastasis by crosstalk with tumor cells through CX3CR1 (51). Cluster M4 showed a significant activation of pathways related to cell proliferation including DNA replicate and cell cycle. Furthermore, M4 also highly expressed proliferation marker (MKI676, TOP2A, PCNA and HMGB2) and known as proliferating TAM (54, 55).

Cluster M2 was enriched in LG bladder cancer, and M2 highly expressed transcription factors (ID1 and ELF3), thereby regulating epithelial cell development and mediating inflammatory signals (56–58). Epithelial makers (KRT18, KRT19, KRT17, KRT7, and CLDN4) were also highly expressed (59), indicating that M2 may be derived from transdifferentiation of epithelial cells in the tumor (60, 61). Moreover, M2 also expressed genes related to inflammation and tissue damage repair (GPX2, AGR2, and AQP3) (62, 63), reflecting an anti-inflammatory-related phenotype.

Cluster M3 was significantly enriched in HG patient. M3 showed a significant activation of cholesterol metabolism pathway and immune-related pathways. Macrophage in this cluster had high expression of type I interferon signaling related genes (IFITM3, IFI27, MX1, IFI6, and ISG15) as well as gene features related to immunosuppressive phenotype (APOE, APOC1, S100A9, and GPNMB) (47, 64, 65), whereby participating in tumor immune regulation (66, 67). Although immunosuppressive genes were expressed in M3 cluster macrophages, SPP1 (OPN) was the most differentially expressed gene in the group of HG bladder cancer-specific macrophages (**Supplementary Figure S3E**). SPP1 was a potent macrophage chemokine (68) that can regulate interferon signaling (69) to inhibit T-cell activation and thus create an immune-tolerant environment (70).

### Endothelial Cells and Fibroblasts Are Enriched in HG Relapse

We then studied endothelial cells and fibroblasts in TME. Both endothelial cells and fibroblasts were enriched in high-grade relapse patients (**Supplementary Figures S4A, S5A**), indicating that stromal cells play an important role in the TME of relapse patients.

**Figure 6:**
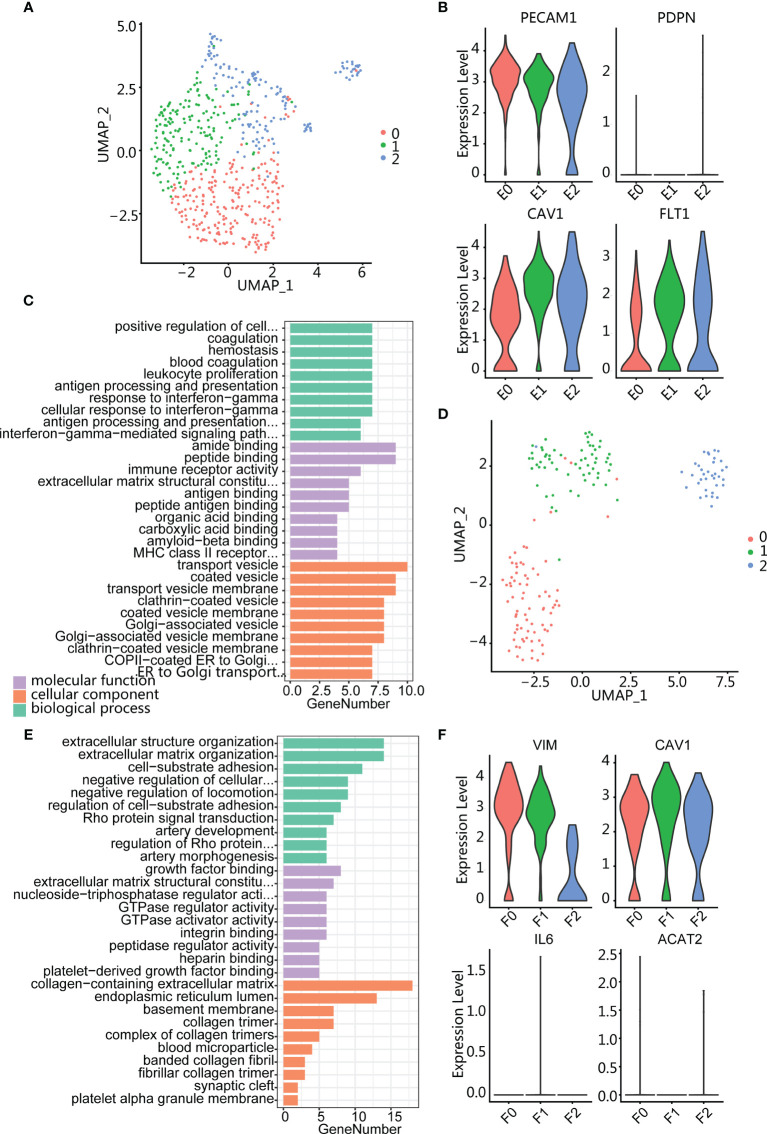
Endothelium and fibroblasts enriched in high-grade relapse cancer. **(A)** UMAP plot of endothelial cells; color indicates cell population subtypes. **(B)** Violin plots showing the expression level of marker genes among endothelial cells. **(C)** Bar plot showing the most enrichment GO terms for E0 cluster. **(D)** UMAP plot of fibroblasts; color indicates cell population subtypes. **(E)** Bar plot showing the most enrichment GO terms for F1 cluster. **(F)** Violin plots showing the expression level of marker genes among fibroblasts.

Three clusters (E0, E1, and E2) were obtained by clustering analysis of endothelial cells (**Figure 6A**). All these clusters highly expressed endothelial marker genes PECAM1 and extracellular matrix protein marker CAV1 (**Figure 6B**). In addition, the three subpopulations all expressed vascular endothelial growth factor marker FLT1 but did not express lymphatic endothelial marker PDPN, indicating that endothelial cells were derived from vascular rather than lymphatic vessels. Next, we performed GO enrichment analysis on the three clusters of endothelial cells and found that E0 cluster was enriched in immune-related pathways such as antigen processing and presentation (**Figure 6C**), while E1 and E2 are enriched in endothelium development, extracellular matrix organization, and regulation of angiogenesis (**Supplementary Figure S4D**), suggesting that the E2 cluster is related to tumor growth and migration.

Fibroblasts were also divided into three clusters (F0, F1, and F2) (**Figure 6D**), and all subpopulations expressed mesenchymal marker VIM, matrix protein marker CAV1, and absence myofibroblast canonical marker ACAT2 (**Figure 6F**). Gene enrichment analysis showed that cluster F0 was enriched in the muscle system process (**Supplementary Figure S5C**) and F2 was enriched in regulated cell growth and extracellular matrix organization (**Supplementary Figure S5D**). Cluster F1 expressed collagen-associated protein genes (COL1A2 and COL6A3) and was enriched in terms of cell-substrate adhesion (**Figure 6E**; **Supplementary S5B**). In addition, F1 also specifically expressed THY1 (**Supplementary Figure S5B**), indicating an activated fibroblast subset and playing an important role in tumor-supported collagen synthesis.

In summary, macrophage infiltration was predominant in LG, HG, and HG relapse bladder cancer microenvironment, rather than T-cell infiltration. The infiltrated T cells and macrophages differed greatly before and after relapse of the HG bladder cancer patient. Both LG and HG bladder cancer exhibited immunosuppressive phenotypic macrophage infiltration, while HG relapse patient were mainly infiltrated by proliferating macrophages and memory T cells. In contrast, the population of immune-activated phenotypic T cells infiltrating in patient with CG increased while the infiltration of macrophages with immunosuppressive phenotype decreased.

## Discussion

In this study, we performed single-cell RNA sequencing of CG, LG, HG, and its recurring bladder samples, producing the single-cell transcriptome data that allowed us to describe the biological characteristics of different clinical state samples. We inferred the CNV from the gene expression profile to determine whether the epithelium was malignant. The accidental discovery that some epithelial cells from CG sample were also judged as malignant indicated that inflammatory lesions also had gene copy number variation. Subsequent analysis found that although copy number variation existed in the CG epithelium, its gene expression profile and microenvironment immune activation phenotype were obviously different from the cancer immunosuppressive phenotype, which was not enough to indicate that it had a tendency to become cancerous.

Simultaneously, we revealed the transcriptome-specific signatures of different clinical conditions. The signatures of N-glycan biosynthesis were specifically enriched into HG bladder cancer, which were significantly associated with poor overall survival. The inflammatory response-related signatures including TNF pathway were mainly enriched in CG, while gene features associated with cell invasion and drug resistance were highly expressed in patient with HG relapse.

N-linked glycosylation is one of the most common protein posttranslational modifications. Under the action of glycosyltransferase, protein-specific amino acid residues will form glycosidic bonds with glycogroups. Abnormal glycosylation is closely related to tumor occurrence, development, and invasion (71), which also explains why HG bladder cancer has a strong ability to recur and become invasive. Oligosaccharyltransferase (OST) is a key enzyme in the N-glycan biosynthesis process. The use of OST inhibitor NGI-1 can inhibit the glycosylation of receptor tyrosine kinases (RTK), thereby inhibiting the proliferation of nonsmall cell lung cancer (72). Our findings demonstrate that OST might be a potential therapeutic target for HG bladder cancer.

Bladder cancer is one of the tumors with the least immune infiltration, and it is also one of the tumors with the worst immune response to checkpoint inhibitors such as PD1 and PDL1 (73). It is necessary to improve the understanding of the immune environment in this patient population. We observed lower T-cell infiltration in the TME, consistent with those previously reported (73). In HG bladder cancer, macrophages with immunosuppressive phenotype were enriched and T-cell infiltration was less. These are all features that are contrary to the environment in which an immune response can be established. These TME changes are dynamic, because we found that more immune memory cell infiltration phenotype and more proliferative macrophage infiltration after patient recurrence. This inflammatory state may have a synergistic effect with the emergence of drug resistance and may result in crosstalk between cancer cells and TME. Therefore, inducing an immunostimulatory phenotype during bladder therapy and providing a supporting TME help prevent recurrence and enhance the antitumor response.

In summary, we have identified additional potential targets of OST in high-grade bladder cancer and described the immune cell composition of bladder cancer microenvironment in different clinical states by making more precise comparisons between different cell types. Our data will provide a basis for the development of new therapies for high-grade bladder cancer, but there are still some limitations to our work. Firstly, the number of samples used is too small and the algorithm for inferring epithelial malignancy needs further improvement. Secondly, the role of mesenchymal cells such as fibroblasts and endothelial cells also needs to be further studied by increasing the number of cells. Finally, our research requires to be further verified by other experiments including immunohistochemistry.

## Data Availability Statement

The datasets presented in this study can be found in online repositories. The names of the repository/repositories and accession number(s) can be found in the article/**Supplementary Material**.

## Ethics Statement

The studies involving human participants were reviewed and approved by The Ethics Committee Board of Luohu District People’s Hospital. The patients/participants provided their written informed consent to participate in this study.

## Author Contributions

TT, SW, and YL conceived and designed the study. YL performed the data analyses and wrote the manuscript. TT, RT, and GH collected samples and sent them to the company for sequencing. TT reviewed and edited the manuscript. All authors read and approved the final version of the manuscript.

## Funding

This study was supported by the National Natural Science Foundation of China (81922046 and 61931024), the Special Funds for Strategic Emerging Industries Development in Shenzhen (20180309163446298), Shenzhen Science and Technology Innovation Commission (RCJC20200714114557005), and Shenzhen Key Laboratory Program (ZDSYS20190902092857146).

## Conflict of Interest

The authors declare that the research was conducted in the absence of any commercial or financial relationships that could be construed as a potential conflict of interest.

## Publisher’s Note

All claims expressed in this article are solely those of the authors and do not necessarily represent those of their affiliated organizations, or those of the publisher, the editors and the reviewers. Any product that may be evaluated in this article, or claim that may be made by its manufacturer, is not guaranteed or endorsed by the publisher.
