# Supplementary material for: Single-Cell Transcriptome Comparison of Bladder Cancer Reveals Its Ecosystem
Source: Front Oncol. 2022 Feb 21;12:818147. doi: 10.3389/fonc.2022.818147 (PMC8899594; doi:10.3389/fonc.2022.818147)
Supplement: Supplementary file 1 [file DataSheet_1.docx]

Supplementary Material

# Supplementary Data

The raw sequence data analyzed in this study have been deposited in the public GEO database under accession number GSE190888 . TCGA BLCA datasets used in this study can be found on this website UCSC XENA (https://xenabrowser.net). The codes used to process sequencing data can be found on github repository at https://github.com/Zhumd/BLCA_scRNA. All other details are available from the corresponding author upon request.

# Supplementary Figures and Tables

## Supplementary Figures


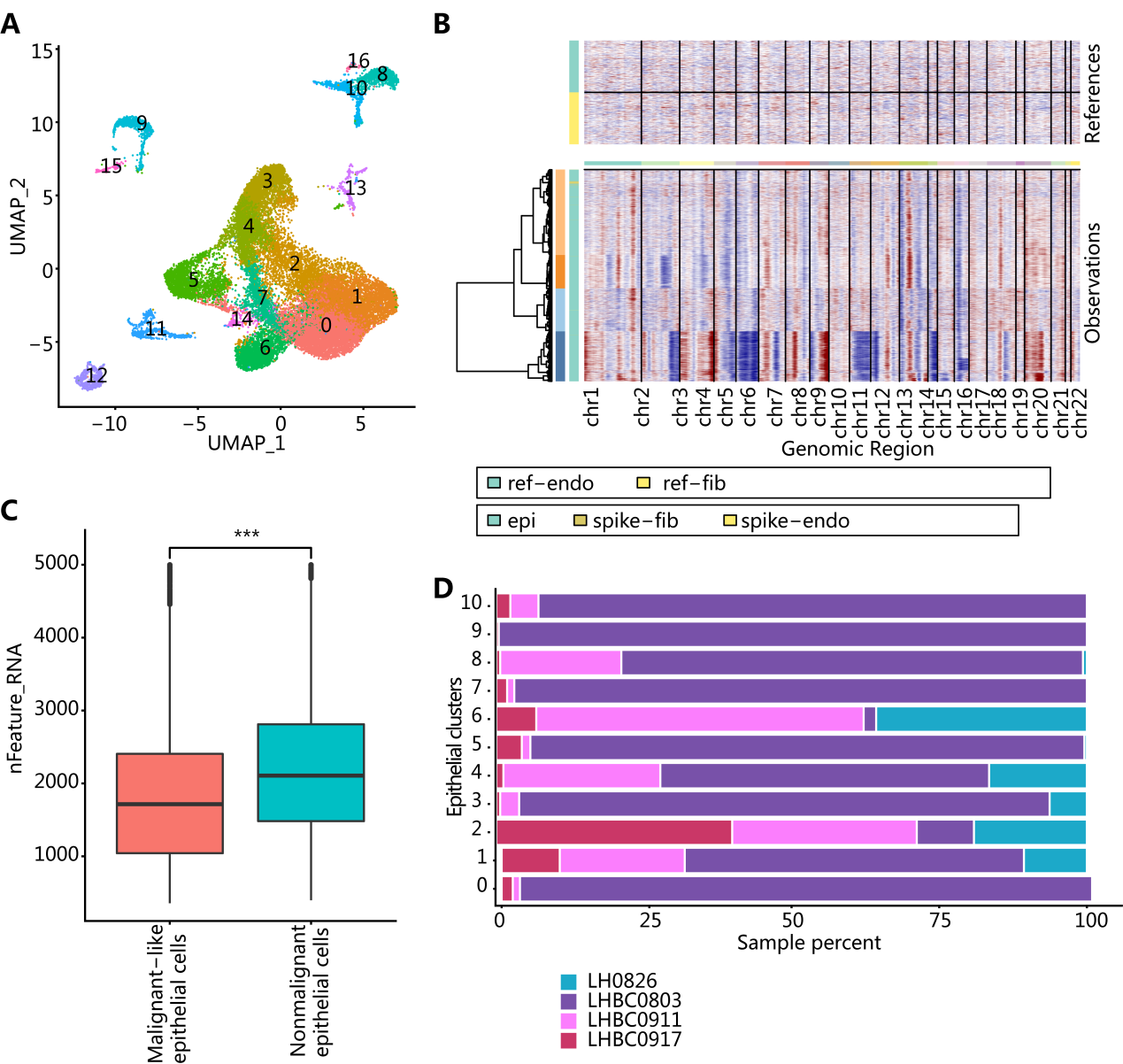


**Supplementary Figure 1.** CNV inference analysis of epithelial cells. **A.** UMAP plot of 17 clusters for all cells, and the color indicates clusters. **B.** Heatmap showed that the inferred CNV helps to identify malignant-like and non-malignant epithelium, with red indicating amplifications and blue indicating deletions. **C.** Boxplot showing the number of genes expressed in malignant-like and non-malignant epithelium. **D.** Bar plot showing the distribution of 11 malignant-like epithelial clusters in different samples.


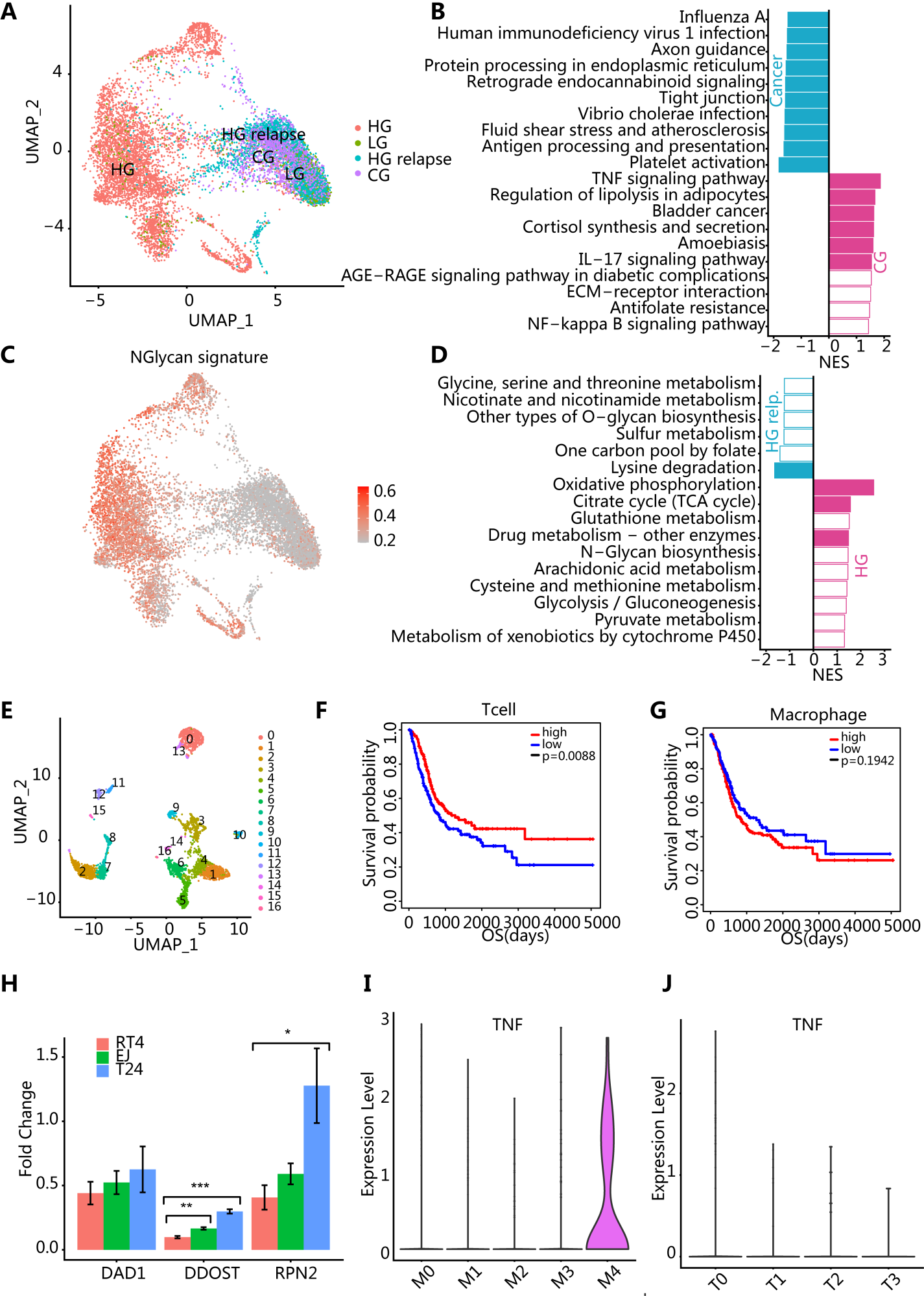


**Supplementary Figure 2.** Analysis of differential gene expression between malignant epitheliums in different clinical states. **A.** UMAP plot of malignant epithelial cells, and the color represents different samples. **B.** Bar plots showing the pathway differentially activated between CG and Cancer. The color indicates significance P < 0.05, and the x-axis is the enrichment fraction. **C.** UMAP plot of malignant-like epithelial cells, color represents N-Glycan signature (the average expression of genes enriched into this pathway). **D.** Bar plots showing the pathway differentially activated between HG and HG relapse. The color indicates significance P < 0.05, and the x-axis is the enrichment fraction. **E.** UMAP plot of 17 clusters of non epithelial cells, and the color indicates clusters. **F**. Kaplan-Meier analysis of T cells with overall patient survival in TCGA dataset. P =0.0088 using log-rank test. **G** Kaplan-Meier analysis of macrophages with overall patient survival in TCGA dataset. P = 0.1942 using log-rank test. **H**. Fold-change expression of OST related genes (DAD1, DDOST and RPN2) as quantified by q-PCR in RT4, EJ and T24 bladder cancer cell line. *P < 0.05, **P < 0.01, ***P < 0.001 using two side unpaired Student's t-test. **I,J**. TNF expression level in macrophage **(I)** or T cell clusters **(J)**.


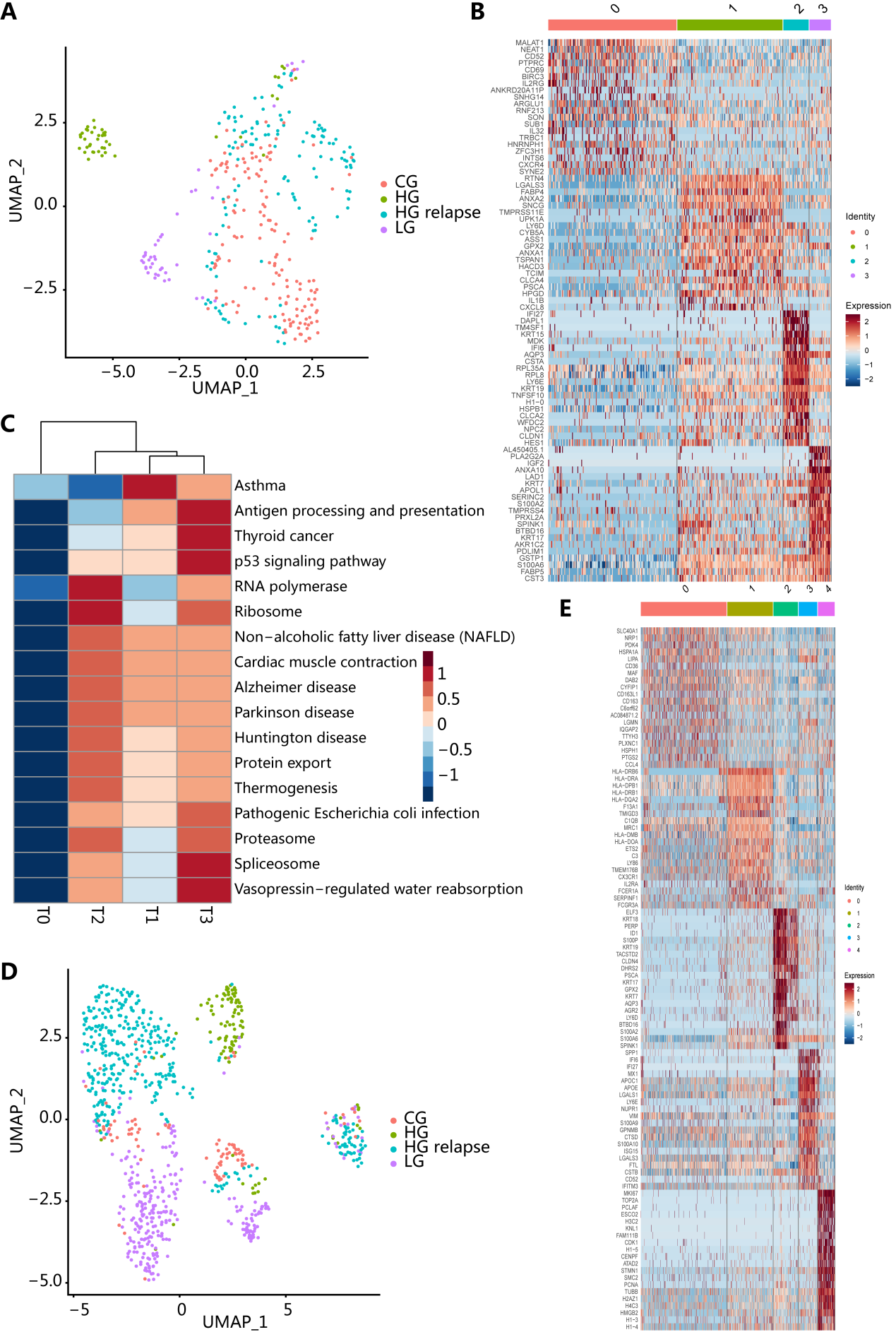


**Supplementary Figure 3.** Analysis of differential gene expression between T cells and macrophages. **A.** UMAP plot of T cells, color indicates different samples. **B.** Heatmap showing the top 20 differentially overexpressed genes in each T cell cluster. **C.** Heatmap of differential activation

pathways for four T cell clusters. **D.** UMAP plot of macrophages, color indicates different samples. **E.** Heatmap showing the top 20 differentially overexpressed genes in each macrophage cluster.


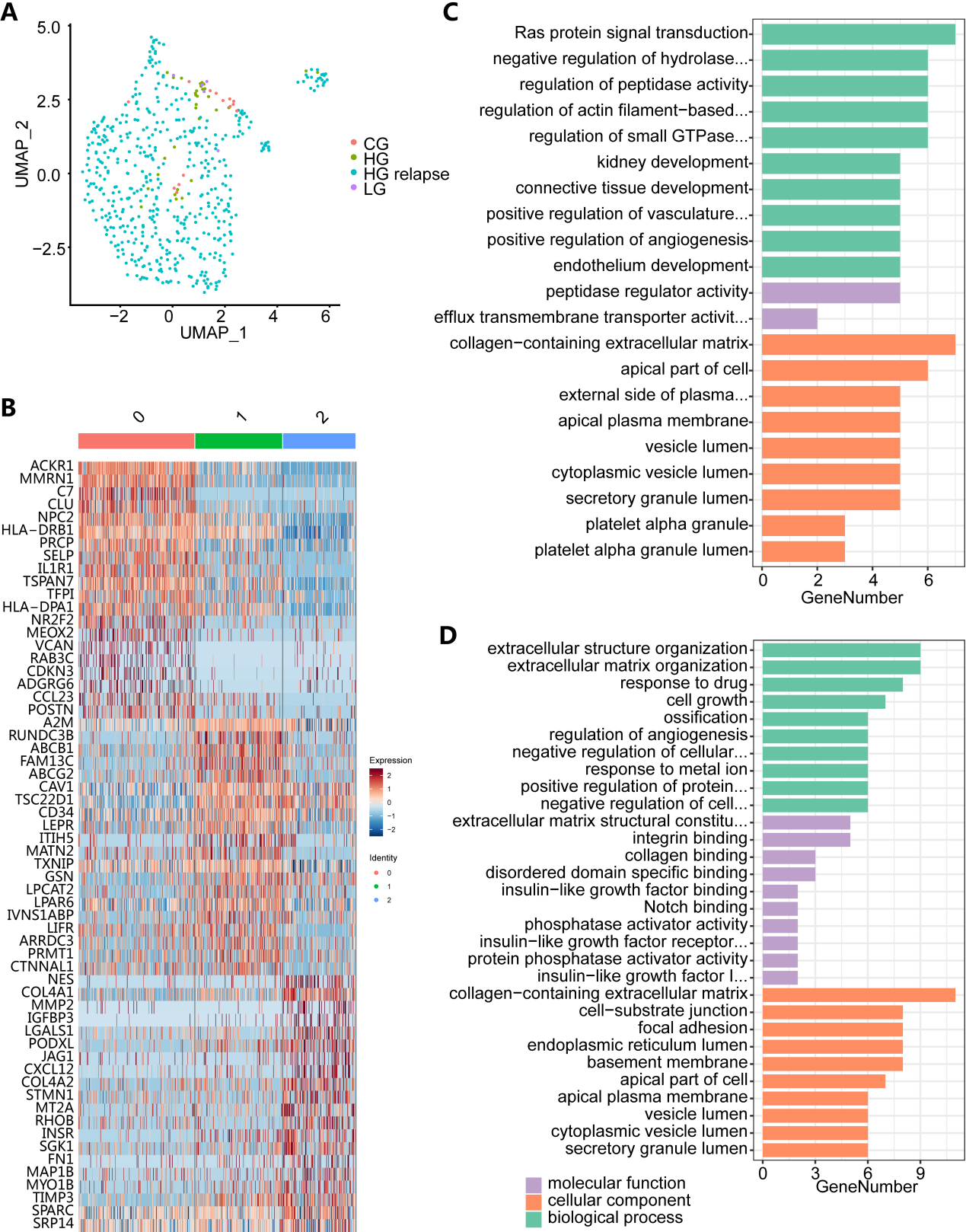


**Supplementary Figure 4.** Analysis of differential gene expression of endothelial cells. **A.** UMAP plot of endothelial cells, color indicates different samples. **B.** Heatmap showing the top 20 differentially overexpressed genes in each endothelial cell cluster. **C,D** Bar plot showing the most enrichment GO terms for E1 **(C)** or E2 **(D)** cluster.


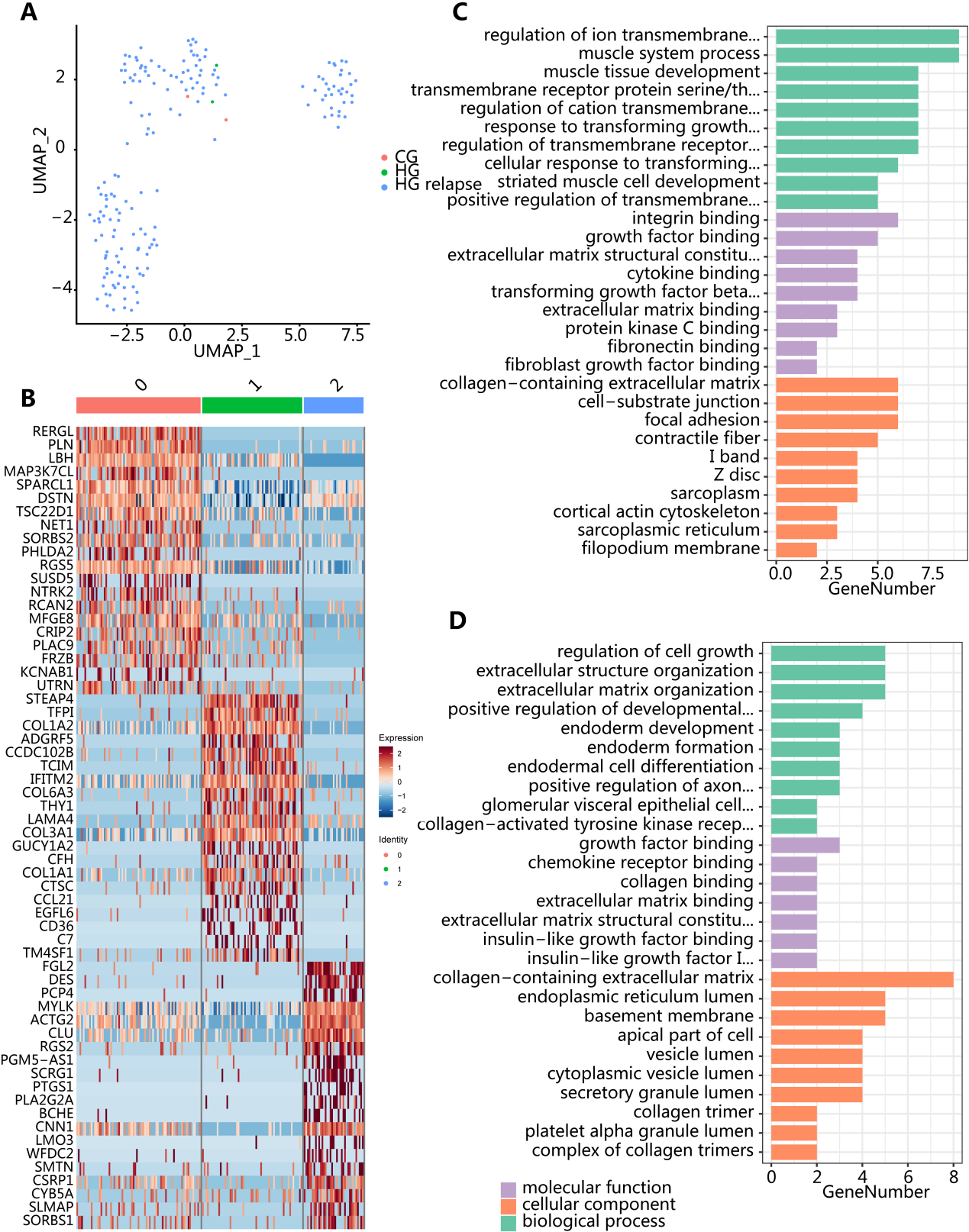


**Supplementary Figure 5.** Analysis of differential gene expression of fibroblasts. **A.** UMAP plot of fibroblasts, color indicates different samples. **B.** Heatmap showing the top 20 differentially overexpressed genes in each fibroblasts cluster. **C,D.** Bar plot showing the most enrichment GO terms for F0 **(C)** or F2 **(D)** cluster.

**2.2 Supplementary tables**

**Supplementary Table 1** Characteristics of patients, some enriched terms and markers used in this study.

| The clinical characteristics of patients included in this study. | | | | | | | | | | | | | | | | | |
| --- | --- | --- | --- | --- | --- | --- | --- | --- | --- | --- | --- | --- | --- | --- | --- | --- | --- |
| **Sample** | | **Type** | | | | | **Grading** | | | | **Age** | | **Gender** | | | **Other** | |
| LHBC0803 | | Urothelial Carcinoma | | | | | High Grade | | | | 58 | | Male | | | None | |
| LH0826 | | Cystitis Glandularis | | | | | None | | | | 39 | | Male | | | None | |
| LHBC0911 | | Urothelial Carcinoma | | | | | High Grade | | | | 58 | | Male | | | LHBC0803 Recurrence | |
| LHBC0917 | | Urothelial Carcinoma | | | | | Low Grade | | | | 28 | | Male | | | None | |
| Cystitis glandularis (CG) GO annotation terms of up expression genes. | | | | | | | | | | | | | | | | | |
| **Category** | | | **Term** | | | **Count** | | | **%** | | **PValue** | | | | **Genes** | | |
| GOTERM_BP_DIRECT | | | GO:0071356~cellular response to tumor necrosis factor | | | 2 | | | 25 | | 0.038672 | | | | FABP4, CXCL8 | | |
| GOTERM_CC_DIRECT | | | GO:0005615~extracellular space | | | 4 | | | 50 | | 0.003597 | | | | PIGR, CXCL8, OLFM4, MUC4 | | |
| GOTERM_CC_DIRECT | | | GO:0070062~extracellular exosome | | | 4 | | | 50 | | 0.028712 | | | | PIGR, FABP4, OLFM4, MUC4 | | |
| High grade relapse patient enrichment GO and KEGG terms. | | | | | | | | | | | | | | | | | |
| **ID** | | **setSize** | | **enrichmentScore** | | | **NES** | | | **pvalue** | | | | **core_enrichment** | | | |
| regulation of lipid metabolic process | | 4 | | -0.83665 | | | -2.09311 | | | 0.001549 | | | | SQLE/CHD9/NCOR1 | | | |
| regulation of cell growth | | 4 | | -0.72908 | | | -1.824 | | | 0.007057 | | | | KIAA1109/IGFBP5/PLCE1 | | | |
| ABC transporters | | 3 | | -0.92208 | | | -1.84577 | | | 0.000909 | | | | ABCC5/ABCC3 | | | |
| The known immune marker genes were used in this study. | | | | | | | | | | | | | | | | | |
| **Gene** | **Cell Type** | | | | **Gene** | | | **Cell Type** | | | | **Gene** | | | | | **Cell Type** |
| PTPRC | Immune Cells | | | | CD2 | | | Tcell | | | | FCER1A | | | | | Dendritic |
| CD3G | Immune Cells | | | | CD3D | | | Tcell | | | | IGLL5 | | | | | Bcell |
| CD3E | Immune Cells | | | | CD3E | | | Tcell | | | | MZB1 | | | | | Bcell |
| CD79A | Immune Cells | | | | CD3G | | | Tcell | | | | JCHAIN | | | | | Bcell |
| BLNK | Immune Cells | | | | MARCO | | | Macrophage | | | | DERL3 | | | | | Bcell |
| CD68 | Immune Cells | | | | CSF1R | | | Macrophage | | | | SDC1 | | | | | Bcell |
| CSF1R | Immune Cells | | | | CD68 | | | Macrophage | | | | MS4A1 | | | | | Bcell |
| MARCO | Immune Cells | | | | GLDN | | | Macrophage | | | | BANK1 | | | | | Bcell |
| CD207 | Immune Cells | | | | APOE | | | Macrophage | | | | PAX5 | | | | | Bcell |
| PECAM1 | Endothelial | | | | CCL3L1 | | | Macrophage | | | | CD79A | | | | | Bcell |
| CD34 | Endothelial | | | | TREM2 | | | Macrophage | | | | LAMP5 | | | | | pDCs |
| VWF | Endothelial | | | | C1QB | | | Macrophage | | | | SHD | | | | | pDCs |
| EPCAM | Epithelial | | | | NUPR1 | | | Macrophage | | | | LRRC26 | | | | | pDCs |
| SFN | Epithelial | | | | FOLR2 | | | Macrophage | | | | PACSIN1 | | | | | pDCs |
| KRT19 | Epithelial | | | | RNASE1 | | | Macrophage | | | | LILRA4 | | | | | pDCs |
| ACTA2 | Fibroblasts | | | | C1QA | | | Macrophage | | | | CLEC4C | | | | | pDCs |
| MCAM | Fibroblasts | | | | CD1E | | | Dendritic | | | | DNASE1L3 | | | | | pDCs |
| MYLK | Fibroblasts | | | | CD1C | | | Dendritic | | | | SCT | | | | | pDCs |

**Supplementary Table 2**. Up regulated genes in CG.

| gene | p_val | avg_log2FC | pct.1 | pct.2 | p_val_adj |
| --- | --- | --- | --- | --- | --- |
| OLFM4 | 0 | 1.865336 | 0.462 | 0.058 | 0 |
| PIGR | 0 | 1.422527 | 0.332 | 0.031 | 0 |
| MTATP6P1 | 0 | 1.104673 | 0.998 | 0.994 | 0 |
| FABP4 | 1.37E-288 | 1.091884 | 0.495 | 0.102 | 5.79E-284 |
| TCIM | 2.69E-269 | 1.190651 | 0.387 | 0.068 | 1.14E-264 |
| MUC4 | 4.82E-233 | 1.069729 | 0.234 | 0.024 | 2.04E-228 |
| DENND2C | 1.27E-224 | 1.160019 | 0.459 | 0.121 | 5.36E-220 |
| ARL14 | 8.85E-164 | 1.237792 | 0.451 | 0.16 | 3.75E-159 |
| CXCL8 | 3.88E-115 | 1.114126 | 0.319 | 0.098 | 1.64E-110 |
| PTGS2 | 5.09E-38 | 0.575937 | 0.329 | 0.185 | 2.15E-33 |
| EDN1 | 3.77E-162 | 0.83641 | 0.276 | 0.055 | 1.60E-157 |
| CXCL2 | 1.97E-131 | 0.646826 | 0.178 | 0.027 | 8.33E-127 |
| CXCL1 | 9.79E-104 | 0.497375 | 0.103 | 0.01 | 4.15E-99 |
| IL1B | 7.98E-79 | 0.373992 | 0.129 | 0.024 | 3.38E-74 |
| MAP2K3 | 1.95E-16 | 0.26049 | 0.184 | 0.111 | 8.27E-12 |

**Supplementary Table 3**. Partially up regulated genes in HG.

| gene | p_val | avg_log2FC | pct.1 | pct.2 | p_val_adj |
| --- | --- | --- | --- | --- | --- |
| HSPB1 | 0 | 2.754732 | 0.885 | 0.597 | 0 |
| TNFSF10 | 0 | 2.511584 | 0.78 | 0.298 | 0 |
| SERF2 | 0 | 2.189286 | 0.831 | 0.484 | 0 |
| DBI | 0 | 2.050571 | 0.678 | 0.108 | 0 |
| COX6C | 0 | 2.26818 | 0.75 | 0.292 | 0 |
| UQCR10 | 0 | 2.19494 | 0.741 | 0.29 | 0 |
| RPS26 | 0 | 2.076609 | 0.843 | 0.561 | 0 |
| SPCS1 | 1.06E-301 | 2.541533 | 0.679 | 0.141 | 4.47E-297 |
| RPS27 | 1.33E-301 | 1.699889 | 0.949 | 0.932 | 5.64E-297 |
| DAD1 | 2.32E-196 | 1.264562 | 0.569 | 0.116 | 9.84E-192 |
| DDOST | 7.64E-146 | 0.919985 | 0.494 | 0.106 | 3.24E-141 |
| RPN1 | 5.43E-99 | 0.737482 | 0.518 | 0.202 | 2.30E-94 |
| RPN2 | 2.44E-63 | 0.623106 | 0.562 | 0.327 | 1.03E-58 |
| DPM3 | 5.42E-90 | 0.555219 | 0.324 | 0.048 | 2.29E-85 |
| DPM1 | 1.41E-73 | 0.516893 | 0.381 | 0.115 | 5.97E-69 |
| DPM2 | 1.16E-68 | 0.497207 | 0.36 | 0.106 | 4.89E-64 |
| ALG3 | 1.08E-71 | 0.482186 | 0.303 | 0.061 | 4.59E-67 |
| MAN1A1 | 3.78E-73 | 0.479243 | 0.242 | 0.021 | 1.60E-68 |

**Supplementary Table 4**. DEG of macrophage clusters.

| **p_val** | **avg_log2FC** | **pct.1** | **pct.2** | **p_val_adj** | **cluster** | **gene** |
| --- | --- | --- | --- | --- | --- | --- |
| 9.04E-14 | 1.555115 | 0.41 | 0.232 | 3.83E-09 | 0 | PDK4 |
| 6.71E-14 | 1.309633 | 0.739 | 0.683 | 2.84E-09 | 0 | SLC40A1 |
| 0.000295 | 1.171166 | 0.512 | 0.536 | 1 | 0 | HSPH1 |
| 1.07E-10 | 1.160385 | 0.305 | 0.147 | 4.52E-06 | 0 | CD36 |
| 1.16E-07 | 1.126439 | 0.313 | 0.198 | 0.004927 | 0 | CD163L1 |
| 0.00043 | 1.031717 | 0.41 | 0.37 | 1 | 0 | PTGS2 |
| 2.27E-06 | 0.999141 | 0.329 | 0.228 | 0.095955 | 0 | AC084871.2 |
| 7.66E-14 | 0.995603 | 0.624 | 0.515 | 3.24E-09 | 0 | NRP1 |
| 2.80E-12 | 0.930024 | 0.65 | 0.564 | 1.19E-07 | 0 | LIPA |
| 1.71E-13 | 0.917765 | 0.781 | 0.764 | 7.23E-09 | 0 | HSPA1A |
| 5.32E-90 | 2.100185 | 0.97 | 0.229 | 2.25E-85 | 1 | HLA-DRB6 |
| 5.68E-58 | 1.429558 | 0.852 | 0.237 | 2.40E-53 | 1 | HLA-DQA2 |
| 1.71E-19 | 1.395234 | 0.606 | 0.288 | 7.25E-15 | 1 | FCER1A |
| 3.65E-36 | 1.343943 | 0.645 | 0.208 | 1.55E-31 | 1 | TMIGD3 |
| 8.23E-62 | 1.146852 | 1 | 0.972 | 3.48E-57 | 1 | HLA-DPB1 |
| 5.39E-48 | 1.142891 | 0.877 | 0.298 | 2.28E-43 | 1 | F13A1 |
| 5.69E-66 | 1.112651 | 1 | 0.989 | 2.41E-61 | 1 | HLA-DRA |
| 2.85E-58 | 1.010109 | 1 | 0.982 | 1.21E-53 | 1 | HLA-DRB1 |
| 1.74E-21 | 0.974324 | 0.448 | 0.134 | 7.36E-17 | 1 | IL2RA |
| 2.04E-24 | 0.94276 | 0.803 | 0.471 | 8.64E-20 | 1 | LY86 |
| 1.29E-24 | 2.864688 | 0.651 | 0.246 | 5.48E-20 | 2 | SPINK1 |
| 1.25E-31 | 2.835282 | 0.606 | 0.163 | 5.29E-27 | 2 | S100A2 |
| 2.99E-68 | 2.824936 | 0.963 | 0.253 | 1.27E-63 | 2 | S100P |
| 7.91E-54 | 2.657475 | 0.688 | 0.121 | 3.35E-49 | 2 | KRT7 |
| 1.68E-67 | 2.517813 | 0.872 | 0.179 | 7.10E-63 | 2 | KRT19 |
| 3.68E-61 | 2.463765 | 0.844 | 0.172 | 1.56E-56 | 2 | DHRS2 |
| 4.07E-57 | 2.338071 | 0.716 | 0.12 | 1.73E-52 | 2 | KRT17 |
| 1.72E-63 | 2.332349 | 0.817 | 0.153 | 7.29E-59 | 2 | CLDN4 |
| 1.16E-55 | 2.267864 | 0.743 | 0.13 | 4.93E-51 | 2 | GPX2 |
| 7.54E-82 | 2.262673 | 0.844 | 0.103 | 3.19E-77 | 2 | ELF3 |
| 4.27E-61 | 4.456333 | 0.627 | 0.051 | 1.81E-56 | 3 | SPP1 |
| 1.09E-51 | 3.329964 | 0.47 | 0.027 | 4.63E-47 | 3 | IFI27 |
| 1.16E-43 | 3.216239 | 0.988 | 0.518 | 4.93E-39 | 3 | APOC1 |
| 2.34E-38 | 2.639816 | 0.94 | 0.434 | 9.91E-34 | 3 | APOE |
| 9.36E-59 | 2.456543 | 0.771 | 0.104 | 3.96E-54 | 3 | IFI6 |
| 2.55E-38 | 2.219156 | 0.988 | 0.706 | 1.08E-33 | 3 | LGALS1 |
| 3.90E-30 | 2.131875 | 0.976 | 0.588 | 1.65E-25 | 3 | GPNMB |
| 1.01E-33 | 2.081745 | 0.59 | 0.109 | 4.27E-29 | 3 | NUPR1 |
| 7.22E-19 | 1.848816 | 0.458 | 0.106 | 3.06E-14 | 3 | CD52 |
| 7.34E-29 | 1.846929 | 0.976 | 0.736 | 3.11E-24 | 3 | CTSD |
| 1.70E-144 | 3.653981 | 0.973 | 0.024 | 7.20E-140 | 4 | MKI67 |
| 5.83E-80 | 3.563052 | 0.84 | 0.081 | 2.47E-75 | 4 | H1-5 |
| 1.33E-125 | 3.40499 | 0.88 | 0.026 | 5.61E-121 | 4 | TOP2A |
| 3.65E-36 | 3.053995 | 0.92 | 0.46 | 1.54E-31 | 4 | H4C3 |
| 2.21E-53 | 2.819778 | 0.96 | 0.279 | 9.35E-49 | 4 | STMN1 |
| 1.00E-40 | 2.762233 | 0.96 | 0.485 | 4.24E-36 | 4 | H2AZ1 |
| 9.74E-74 | 2.5968 | 0.8 | 0.072 | 4.12E-69 | 4 | CENPF |
| 5.17E-111 | 2.456326 | 0.827 | 0.028 | 2.19E-106 | 4 | PCLAF |
| 3.56E-43 | 2.430948 | 1 | 0.49 | 1.51E-38 | 4 | TUBB |
| 4.68E-35 | 2.186857 | 0.92 | 0.378 | 1.98E-30 | 4 | HMGB2 |
